# Collaborating to offer HPV vaccinations in jails: results from a pre-implementation study in four states

**DOI:** 10.1186/s12913-021-06315-5

**Published:** 2021-04-07

**Authors:** Amanda Emerson, Molly Allison, Lisa Saldana, Patricia J. Kelly, Megha Ramaswamy

**Affiliations:** 1 School of Nursing and Health Studies, University of Missouri-Kansas City, 2464 Charlotte Street, Kansas City, MO 64108 USA; 2 College of Veterinary Medicine, Kansas State University, Manhattan, KS 66506 USA; 3 Oregon Social Learning Center, 10 Shelton McMurphey Blvd., Eugene, Oregon 97401 USA; 4 Jefferson School of Nursing, Thomas Jefferson University, Center City Campus, 901 Walnut Street, 8th Floor, Philadelphia, PA 19107 USA; 5 Department of Population Health, University of Kansas Medical Center, 3901 Rainbow Boulevard, Kansas City, KS 66160 USA

**Keywords:** Human papillomavirus, Vaccination, Prisoner populations, Health departments, Interagency collaboration

## Abstract

**Background:**

Correctional facilities are an underutilized venue for reaching young adults who have not vaccinated for human papillomavirus (HPV). The objective of this study was to identify factors that are associated with jail and local health department (LHD) interest in partnering to offer HPV vaccinations to young adults in jail.

**Methods:**

Consolidated framework for implementation research (CFIR)-guided surveys were conducted with jail administrators in Iowa, Kansas, Missouri, and Nebraska, September 2017–October 2018. Jail survey data were analyzed using chi square distribution and relative risk regression. Using data from sister surveys conducted with LHD administrators in the same counties (results previously reported), we identified characteristics of counties in which both the jail *and* LHD indicated interest in collaborating to offer HPV vaccinations in the jail.

**Results:**

Jail survey response was 192/347 (55.3%). Surveys with LHDs yielded 237/344 (68.9%) responses. Eleven communities were identified where both the jail and LHD expressed interest. Only “any vaccines provided in jail” predicted shared interest (RR: 5.36; CI: 2.52–11.40; *p* < .01). For jail administrators, offering other vaccines was 3 times (CI:1.49–6.01; *p* < .01) and employing a nurse 1.65 times more likely (CI: 1.20–2.28; *p* < .01) to predict interest in collaborating to offer HPV vaccination. Open-ended responses indicated that managing linkages and stakeholder investment were areas of emphasis where collaborations to provide vaccinations in the jails had been previously implemented.

**Conclusions:**

Interest in jail-LHD partnerships to provide HPV vaccinations in jails exists in the Midwest but will require building-out existing programs and linkages and identifying and strengthening shared values, goals, and benefits at all levels.

**Supplementary Information:**

The online version contains supplementary material available at 10.1186/s12913-021-06315-5.

## Background

Newly diagnosed *human papillomavirus*-associated cancers in the United States number approximately 34,800 per year and cost an estimated $1.7 billion [1, 2]. The highest rates of HPV infection occur in young adults, a group that initiates HPV vaccination at comparatively lower rates than other recommended groups [2, 3]. The four-state Department of Health and Human Services (DHHS) Region VII (Iowa, Kansas, Missouri, Nebraska) ranks eighth of ten regions for HPV vaccination coverage [4]. Kansas and Missouri rank in the lowest quartile of states based on most recent National Immunization Survey data [4].

This study focused on the vaccination needs of young adults, ages 19–26, who move through jails in DHHS Region VII. Justice-involved young adults are often exposed to social determinant and lifestyle factors that are linked to increased risk of developing and dying from HPV-related cancers—e.g., racial minority status, smoking, poverty, childhood sexual abuse, early sexual debut, and multiple sex partners [5–7]. We have known for some time that women incarcerated in jail are 4–5 times more likely than community samples to have been diagnosed with cervical cancer [8]. Persons with a history of low-level, repeated incarcerations are more likely to be overlooked for vaccination outreach than others, as we learned in previous research, where only 21% of vaccine eligible adults (*n* = 80) in a urban Kansas jail reported receiving any HPV vaccine, and a little over half were unaware that such a vaccine existed [9]. Seventy percent (*n* = 181) reported never receiving a provider’s recommendation for HPV vaccination [10]. Poverty, lack of health insurance, and provider hesitancy stemming from reluctance to discuss or misconceptions about the efficacy of the vaccine for this group make HPV vaccination among justice-involved young adults especially challenging [5, 9, 11].

### Looking for solutions: bringing vaccines to incarcerated adults

Jails are not obvious venues for preventive health care, yet from a public health perspective they offer a potentially high impact opportunity to reach an underserved population [12]. Compared with the general population, incarcerated persons tend to have poorer health and less access to health services [13, 14]. Outside large urban systems, preventive services like vaccination are rarely provided in jails [15]. In smaller, rural communities, the local health department—also under county jurisdiction in most states—is a primary source of HPV and other vaccinations for low-income adults [16]. Bringing jails and local health departments together to offer vaccinations in jails is one way to extend access to preventive services to a justice-involved population that may otherwise remain overlooked and underserved. Currently, not much is known about what it would take to facilitate jail-LHD collaborations.

In this study, we sought to learn what elements relevant to interest and readiness to collaborate across agencies could be identified in jails and, further, what distinguished counties in which both jail and LHD administrators expressed interest in collaborating to offer HPV vaccinations in the jail. Our results may help guide future efforts to bring agencies together to expand HPV protection in a high-risk population and possibly offer other vaccinations and preventive health services as well.

## Methods

### Design, sample, and setting

The primary sample for this cross-sectional survey study was jail administrators in 347 counties in Kansas, Missouri, Iowa, and Nebraska. Surveys were also conducted with LHD administrators in the same communities and those results have been published previously [17]. Both the jail and LHD administrators were identified using internet searches, state directories, and research contacts, and were recruited and surveyed via email between November 2017 and October 2018. Jails included state jails, county jails, municipal jails, and municipal-county jails (i.e., unified city and county). The eligibility criterion was geographic location in a Region VII state. Of the 412 counties in Iowa, Kansas, Missouri, and Nebraska, 62 were excluded because they contracted for jail services with other counties; three were excluded because they had no geographically-associated local health department. The LHD surveys were conducted separately in the same Region VII counties, following the same procedures [17] Since the LHD results have been previously published, only the data collection and results for the jail administrator surveys and the analysis for the combined regression are reported below. The Institutional Review Board at the University of Kansas Medical Center approved the study protocol.

### Data collection

Surveys were sent to jail administrators, beginning in November 2017. The emails were addressed to persons identified in directories or facility web sites as sheriff, or a comparable administrative leadership role. After 1 week, a member of our study staff attempted to make phone contact with administrators who did not return a survey and offered to conduct the survey by telephone, fax, or, where feasible, in person. We made three attempts to reach administrators. The open-ended questions asked about already implemented or planned programs. The open-ended questions were posed only to those who answered positively to the survey question about having already implemented vaccination in the jail through a collaboration. These were invited via email and the questions were administered via phone by a trained Master of Public Health student who took notes and read back responses to verify accuracy.

### Measures

The jail administrator survey, like the nearly identical health department survey, was created by the authors for this study and was based on CFIR domains [18] and the results of a previously published focus group study that we conducted with six jail administrators and seven LHD administrators [19]. The survey development is discussed in a supplemental file published with the article in which we reported on results from the LHD surveys [17]. Copies of the surveys themselves (jail and LHD) are available as Additional files 1 and 2 in the online version of this article. The jail survey elicited information about our primary dependent variable, interest in collaborating with the LHD to offer HPV vaccinations to adults during a jail detention. This question read: “How ready is your facility for implementing an HPV vaccination program with the help of a local health department?” Respondents could choose from among three positive responses (“Already implemented,” “Interested and has some groundwork laid to implement,” and “Interested in learning more about implementation”) and two negative responses (“No interest or intention to implement,” “Don’t know/Declined”). The question corresponded with CFIR domains 3 (inner setting—readiness for implementation) and 4 (characteristics of individuals—individual stage of change) [18].

Other questions addressed facility size and number of staff, jail admissions per year, health care services available, and types of providers staffed. The survey assessed perceptions about priority and perceived suitability of offering vaccinations; priority health care needs of the detained adult population; and potential challenges and opportunities for collaborating to offer HPV vaccine, including security concerns, cost, and logistics related to communication, space, and documentation/recordkeeping. The 12 open-ended questions about initiation, development, facilitators, and impediments of already implemented programs appear in Additional file 3.

### Data analysis

#### Jail administrator survey

We calculated frequencies for categorical variables and means, medians, and standard deviations (SD) for continuous variables and used chi-square tests for goodness of fit to evaluate differences in proportions. Characteristics of the jails and interest in an HPV vaccination program were analyzed first by state to determine whether state-level variations existed that might affect future intervention design and implementation. We used modified Poisson relative risk regression to evaluate associations between the dependent variable (jail interest) and characteristics of the jails. The dependent variable of interest was dichotomized as positive: 0 = already implemented; interested/has groundwork; and interested in learning more; or negative: 1 = no interest or don’t know. Median cut points were used to dichotomize continuous measures. Counties were coded either as metropolitan or nonmetropolitan based on designations of the National Center for Health Statistics which categorizes statistical areas based on population center sizes, population density, and community characteristics [20]. Variables not meeting the confidence threshold (*p* ≥ 0.05) were eliminated stepwise from the model until an optimal set was estimated. All analyses were conducted in SAS version 9.4.

#### Shared jail-LHD interest in collaborating to offer HPV vaccinations

Using matched data from our previously reported health department administrator surveys (*n* = 237) [17] and the jail survey data from this study, we also compared counties where the jail and LHD both expressed interest with counties where there was no shared interest in order to identify which variables were associated with having shared interest. After removing variables of least significance and those for which there was no correspondence (i.e., questions in one survey with no matches in the other), we applied modified Poisson relative risk regression to estimate a best-fit model for counties with the shared interest.

#### Open-ended questions

The first author coded CFIR domains and constructs [18] in the open-ended responses and used content analysis techniques to reduce the patterns to themes [21]. The themes and their application were reviewed by all authors, with consensus about themes achieved through discussion. Themes were brought together with the survey results to help reflect on how barriers and facilitators identified in the surveys might play out in actual implementation.

## Results

### Characteristics of responding jails

We received surveys from 192/347 (55%) jails in Iowa (*n* = 26), Kansas (*n* = 70), Missouri (*n* = 58), and Nebraska (*n* = 38). T-tests did not show significant differences between responding and non-responding jails by state. The majority of jails were county-administered (*n* = 165; 85.9%) or combined county-municipal entities (*n* = 26; 13.5%). One state facility responded. Responding counties were mostly nonmetropolitan or rural (*n* = 138; 74.5%), and 99 of the nonmetropolitan were also noncore (i.e., having no anchor community with a population density > 10,000 persons). These results are in keeping with the rural profile of the majority of counties in the Region VII states.

Our primary outcome variable was captured in a question that tapped constructs of readiness for implementation from the CFIR domains 3 (inner setting) and 4 (characteristics of individuals) by querying administrators’ interest in collaborating to provide HPV vaccine (see survey; Additional file 1). The 45 positive responses (dichotomized as “yes”) included “Already have a program” (*n =* 1); “Interested and have some groundwork laid to implement” (*n =* 1); and “Interested in learning more about implementation” (*n =* 43). Negative responses (dichotomized as “no”) included “No interest or intention to implement” (*n =* 90) and “Don’t know/Declined” (*n =* 54). Thirty-six jails indicated that they had ever offered any vaccinations to persons incarcerated in jail, but only four indicated HPV vaccine.

### Jail interest in offering HPV vaccination

Chi-square, bivariate analysis detected significant (*p* < .05) relationships between jail administrators’ interest in offering HPV vaccine and six independent variables: (a) having a registered nurse on staff at the jail, (b) providing any vaccines in the jail, (c) concerns regarding cost, (d) having a medical clinic in the jail where vaccines could be administered, (e) *not* answering “Don’t know” to the question about whether there was space in the jail where vaccine could be administered, and (f) offering treatment for sexually transmitted infections (STIs) in the jail.

Poisson relative risk regression, which included data only for respondents who provided an answer to the readiness/interest question (*n* = 141), yielded a model with two factors remaining significant: any vaccines already provided to inmates (CI: 1.49–6.01; *p* < .01) and having a registered nurse on staff (CI: 1.20–2.28; *p* < .01) (Table 1). Jails in communities with a history of offering vaccines to detainees were 3 times more likely to be interested in partnering with health departments to offer HPV vaccine than jails that had no previous or existing program. Jails with a registered nurse (RN) on staff were 1.65 times more likely to affirm interest in collaboration than jails with no RN on staff.

**Table 1:** Relative risk regression for predictors of jail interest in collaborating to offer HPV vaccination

Independent Variable	Risk Ratio	95% Confidence Interval	*p*-value
Having a registered nurse on staff	1.65	1.20–2.28	< 0.01
Providing any vaccines in the jail	3.00	1.49–6.01	< 0.01

### Shared jail-LHD interest in offering HPV vaccination

Eleven counties in the four-state region demonstrated shared jail-LHD interest in offering vaccinations in the jails: Missouri (5), Kansas (3), Nebraska (2), and Iowa (1). Shared interest was found most frequently in counties that were nonmetropolitan (*n* = 9) but also included two large fringe metropolitan counties—Douglas County, Nebraska, and St. Louis County, Missouri. Neither state nor metropolitan/nonmetropolitan designation was a significant variable for shared interest.

The sole factor in the model predictive of shared interest was vaccines already provided in the jail (RR = 5.36, CI: 2.66–11.94, *p* < .01) (Table 2).

**Table 2:** Relative risk regression for predictors of shared (jail-LHD) interest in collaborating to offer HPV vaccination

Independent Variable^a^	Risk Ratio	95% Confidence Interval	*P*-value
Urbanization (metropolitan county)	0.45	0.19–1.30	0.16
Any vaccines provided to inmates	5.36	2.52–11.40	< 0.01
STI treatment offered	1.73	0.78–3.82	0.18

^a^Variables of least significance removed

### Open-ended responses

Of 36 administrators who responded that they had already implemented or had groundwork to implement vaccinations through a collaboration, five administrators in five different counties provided further information through open-ended follow-up questions. Respondents were health department administrators in Kansas (1) and Missouri (3) and one jail administrator in Missouri. Two were from counties where the jail and LHD collaborated to offer HPV vaccinations in the jail. The other three were from counties where collaborations had been formed to offer influenza, tetanus, hepatitis A, or hepatitis B vaccinations.

Two patterns identified in the open-ended responses were *managing linkages* and *stakeholder investment*. Managing linkages reflected CFIR outer and inner setting domains and was exemplified in responses that described established lines of interagency communication, resource-sharing, reciprocal programming, and physical contiguity or proximity of the participating entities. These emphases helped unpack the survey finding that the greatest predictor of shared interest was existence of a previous program. In one community in which an LHD already provided HPV vaccinations in the jails, the LHD administrator, when asked to describe the advent of the program, sketched a complex but apparently workable coordination of staffing and resources across agencies. The program came about originally because the health department had vaccine on hand that was about to expire and, in the LHD administrator’s account, there were no takers in the community. With the jail physically proximate (“caddy-corner”) to the health department, the LHD administrator, who already helped coordinate tuberculosis (TB) testing in the jail, called the nurse who worked in the jail who then “checked with inmates to see if anyone wanted it.” Communications were facilitated by proximity, precedent, and a desire to conserve resources by sharing them. In another community, where the jail was “just a few blocks” away, the LHD administrator described a “strong working relationship” in which LHD nurses provided services in the jail, including screening for STIs and providing hepatitis A vaccinations. The open-ended responses provided detail about how facilitators could be leveraged and barriers to collaboration offset by strategically managing communication, sharing resources, and enhancing perceptions of mutual benefit, especially in situations where LHDs and jails were physically near and relationships between administrators and staff already established.

Stakeholder investment was the second prominent theme in the open-ended responses. Stakeholder investment was related to CFIR domains of inner setting and individual characteristics. We learned that, in several communities where vaccination programs had been implemented in a jail (3 of the 5), the sheriff played a central role in launching the partnership. A sheriff in one community initiated a collaboration following a routine interagency needs assessment meeting with an LHD administrator. In another community, a state-level vaccination coordinator spearheaded a jail-LHD program to offer vaccinations in the jail. Importantly though, initial facilitation from the top was not enough to sustain a program when nurses, guards, and others were not ready to lend support. One LHD administrator described enthusiastic endorsement by the county sheriff followed by a frustrating and fruitless months’ long effort to get staff members at the jail to schedule a nurse’s visit. The closed survey results showed that a jail’s interest in partnering with an LHD was significantly associated with having a nurse on staff; indeed, nearly all who provided open-ended responses referred to the facilitating role of either a jail or LHD nurse who coordinated and performed the vaccinations. Stakeholder investment more generally highlighted the importance of creating and maintaining multilevel engagement—among nurses, sheriffs, guards, county- and state-level vaccination coordinators, and, though noted to a lesser extent, persons detained in the jails.

## Discussion

A focus on HPV vaccination in jails is uncommon [11, 22]—few investigations have been made into implementing vaccinations in correctional facilities at all. This could be because, as our own and other research has shown, jail administrators tend to identify substance abuse and mental health as the top health care priorities in their facilities, not preventive or routine health services [23]. Only 8% of jail administrators in this study indicated that “lack of regular health care” was the top priority health care need for persons detained in their jails. Yet, the literature abounds with evidence that incarcerated persons suffer disproportionately from a range of chronic and infectious conditions—hypertension, diabetes, asthma, TB, human immunodeficiency virus (HIV), hepatitis C virus (HCV), HPV—requiring preventive, routine, acute, and maintenance care [13, 14, 24].

Our primary result indicated that having any history of offering vaccinations in the jails predicted a community’s having a shared (jail-LHD) interest in collaborating to offer HPV vaccine in the jails. The open-ended responses gave some insight into why this variable, corresponding to CFIR inner-setting construct readiness for implementation, might be so important. Respondents described how barriers to collaboration were fewer when systems were in place to manage linkages by facilitating communication, (re) allocating resources, and promoting staff perceptions of mutual goals and benefits. Other researchers have reported similar findings about the role of pre-existing interagency connections. A group at the University of Nebraska Medical Center in Omaha, Nebraska, credited an established 6 years’ partnership between the university medical center, Omaha public health department, and the Omaha jail with facilitating implementation of their STI education and testing program for persons entering and leaving jail [25]. Similarly, Corcoros, Nettle, and Church cited a 15-year HIV-testing partnership between the Barnstable County, Massachusetts, jails and LHD as contributing to the success of their collaborative HCV screening study in the Cape Cod jails [26]. Lee et al. found that having pre-established interagency linkages between jails and health departments facilitated communication and contributed significantly to the availability of influenza vaccine in smaller jails [27]. Lobato, Roberts, Bazerman, and Hammett’s national survey of jail-LHD collaborations in TB control (e.g., screening at admission, referrals at discharge) in large jails showed that the greater the number of pre-existing points of collaboration between jails and health departments the more positive either agency’s perceptions of their interagency TB control collaborations [28].

We found from the surveys that having nurses on staff in a jail was significantly related to jail administrators’ receptivity to collaboration, which corresponded with responses in the open-ended questions that pointed to the importance of obtaining buy-in at multiple organizational levels. Costumbrado, Stirland, and Cox’s intervention pilot of a collaborative program between the LHD and jail to provide HCV testing in the Los Angeles county jail yielded similar findings [29]. Essential to that implementation was comprehensive (top-to-bottom) stakeholder engagement, including “custody personnel (i.e., Sheriff’s deputies, custody assistants, volunteers, and support staff), public health program partners, and medical staff” [29] (p. 6881). The importance of nurses as stakeholders may also reflect the likelihood that administrators in jails with registered nurses on staff would be more apt to recognize vaccinations as lying within their purview than those without nurses.

Data in this study were self-reported, thus susceptible to selection bias toward an interest in health services programming for persons during incarceration. Our merging of jail and LHD survey data to estimate a model for positive, shared interest in a collaboration was limited by differences in items on the two surveys, so that some points of comparison were either inexact or were unique to one or the other and had to be omitted from the matched analysis. While we had adequate power to detect effects in the jail surveys, the very small sample in the shared interest analysis (*n* = 11) could have constrained our ability to detect relationships. The open-ended responses were helpful to us in conceptualizing how the quantitative findings might play out in the field, but they also garnered very few responses. That there was general consistency in results from the surveys, our preliminary case study [19], and the related literature strengthened our confidence in the generalizability of results.

## Conclusions

Over 10 million jail admissions are logged in the US each year. Persons with a history of incarceration struggle with chronically poor health and life situations that put them at high risk for STIs like the cancer-causing HPV. Jails present a unique opportunity for prevention, though one rarely taken, since most jails provide only minimal health services. Interagency collaborations offer a promising way to extend services to groups who may otherwise be difficult to reach, particularly in smaller, rural communities. In the case of HPV vaccination, our pre-implementation survey of jail and LHD administrators in counties in four Midwestern states indicated that virtually none offered HPV vaccinations in the jails—though about a third indicated interest. Administrators in counties where any vaccinations had been provided through a jail-LHD partnership provided further insight into how existing programs and available, qualified clinicians can facilitate implementation. Implementation planning should concentrate on fostering connections between agencies. This would require purposeful leveraging of historical and existing programs and development of worker commitment at all levels. Successful jail-LHD partnerships could increase access to HPV vaccination for an underserved population, potentially preventing cancers, saving lives, and contributing to a more equitable culture of health.

## Supplementary Information

**Additional file 1.** Jail Survey about Health and Vaccine Services.

**Additional file 2.** Health Department Survey about Collaborating with Local Jails for HPV Vaccination.

**Additional file 3.** Open-ended Questions.

## Data Availability

Data collected in the study are available from the fourth author on reasonable request.

## Abbreviations

CFIR: Consolidated framework for implementation research
CI: Confidence interval
DHHS: Department of Health and Human Services
HCV: Hepatitis C virus
HIV: Human immunodeficiency virus
HPV: Human papillomavirus
LHD: Local health department
RN: Registered nurse
RR: Relative risk
SD: Standard deviation
STI: Sexually transmitted infection
TB: Tuberculosis
