# Supplementary material for: Collaborating to offer HPV vaccinations in jails: results from a pre-implementation study in four states
Source: BMC Health Serv Res. 2021 Apr 7;21:309. doi: 10.1186/s12913-021-06315-5 (PMC8028758; doi:10.1186/s12913-021-06315-5)
Supplement: Supplementary file 1 — Additional file 1. Jail Survey about Health and Vaccine Services. [file 12913_2021_6315_MOESM1_ESM.pdf]

Jail Survey about Health and Vaccine Services

Thank you so much for participating in this survey about concerns and ideas you may have about helping incarcerated young adults (aged 18-26) and youth (aged 9-17) access the human papillomavirus (HPV) vaccination series. As a reminder, the HPV vaccine prevents several kinds of cancer, and is recommended to be offered with other routine vaccines. We are trying to assess if partnerships between county jails and health departments might serve the HPV vaccine needs of inmates.

This survey should take about 15 minutes. It is anonymous. You can stop at any time or decline to answer any questions.

Thank you!

In which state is your health department located? (Select one answer)

- ◯ Iowa
- ◯ Kansas
- ◯ Missouri
- ◯ Nebraska
- ◯ Declined to answer

In which county is your facility located? __________________________________

Describe your facility:

- ◯ County facility
- ◯ City facility
- ◯ County/city facility combined
- ◯ State facility
- ◯ Declined to answer

What is the capacity of your detention facility? (If none, please specify "0")

Beds for ADULT females: ____________________________

Beds for JUVENILE females: ____________________________

Beds for ADULT males: ____________________________

Beds for JUVENILE males: ____________________________

How would you describe the entity that provides medical care at your facility? (Select one answer)

- ◯ Medical care corporation
- ◯ Partnership with a local university medical system
- ◯ Partnership with a local health system (not a university)
- ◯ Run by a local provider hired by the jail
- ◯ Other, please explain:
- ◯ Declined to answer

If other, please explain:

____

Are medical services at your facility accredited? (Select one answer)

- ◯ No
- ◯ Yes, by the National Commission on Correctional Health Care
- ◯ Yes, by the American Correctional Association
- ◯ Yes, by some other association, please explain:
- ◯ Declined to answer

Please explain:

____

How is health care provision coordinated between adult and juvenile detention systems? (Select one answer)

- ◯ No relationship
- ◯ Shared health expenses
- ◯ Other, please explain:
- ◯ Declined to answer

Please explain:

____

How many days per month (0-30) is routine medical care available with these providers on site at your facility? (If none, please specify "0")

General physicians: ______________________ (DAYS PER MONTH)

OBGYN: ______________________ (DAYS PER MONTH)

Women's health nurse practitioner: ______________________ (DAYS PER MONTH)

Women's health physician's assistant: ______________________ (DAYS PER MONTH)

Certified nurse midwife: ______________________ (DAYS PER MONTH)

Mental health providers: ______________________ (DAYS PER MONTH)

Other specialists (please specify below): ______________________ (DAYS PER MONTH)

Please specify other specialists: ______________________

General nurse practitioner: ______________________ (DAYS PER MONTH)

Physician's assistant: ______________________ (DAYS PER MONTH)

Registered nurse: ______________________ (DAYS PER MONTH)

LPN (licensed practical nurses) or equivalent: ______________________ (DAYS PER MONTH)

Medical assistant (non-licensed): ______________________ (DAYS PER MONTH)

How many of each type of provider is available throughout the week at your facility? (If none, please specify "0")

General physicians: ______________________

OBGYNs: ______________________

Mental health providers: ______________________

Nurse practitioners/ Physician's assistants: ______________________

Registered nurses: ______________________

LPNs (licensed practical nurses) or equivalent: ______________________

Medical assistants (non-licensed): ______________________

Other specialists, please specify below: ______________________

Please specify: ______________________

Does your facility bill third-party payers for health services? (Select one answer)

- ◯ Yes
- ◯ No
- ◯ Do not know
- ◯ Declined to answer

How many exam rooms do you have for medical services? ______________________

How are medications dispensed? (Select all that apply)

- ☐ Through a central medication dispensary window
- ☐ On a medication cart that is brought to each housing unit
- ☐ Other, please specify:
- ☐ Declined to answer

Please specify: ______________________

Please list the top three health issues of inmates in your facility.

Most important:

- ◯ Lack of health insurance in community
- ◯ Lack of regular health care
- ◯ Lack of primary care provider
- ◯ Lack of vaccinations
- ◯ Lack of health knowledge
- ◯ Substance use
- ◯ Mental health issues
- ◯ Chromic disease (example: asthma, diabetes, or heart disease)
- ◯ Infectious disease (example: HIV, sexually transmitted infections, or Hepatitis C)
- ◯ Pregnancy
- ◯ Homelessness
- ◯ Poverty
- ◯ Food/nutrition (including obesity/hunger)
- ◯ Low education
- ◯ Other, please specify:

Please specify other: ______________________________

Second most important:

- ◯ Lack of health insurance in community
- ◯ Lack of regular health care
- ◯ Lack of primary care provider
- ◯ Lack of vaccinations
- ◯ Lack of health knowledge
- ◯ Substance use
- ◯ Mental health issues
- ◯ Chromic disease (example: asthma, diabetes, or heart disease)
- ◯ Infectious disease (example: HIV, sexually transmitted infections, or Hepatitis C)
- ◯ Pregnancy
- ◯ Homelessness
- ◯ Poverty
- ◯ Food/nutrition (including obesity/hunger)
- ◯ Low education
- ◯ Other, please specify:

Please specify other: ______________________________

Third most important:

- ◯ Lack of health insurance in community
- ◯ Lack of regular health care
- ◯ Lack of primary care provider
- ◯ Lack of vaccinations
- ◯ Lack of health knowledge
- ◯ Substance use
- ◯ Mental health issues
- ◯ Chromic disease (example: asthma, diabetes, or heart disease)
- ◯ Infectious disease (example: HIV, sexually transmitted infections, or Hepatitis C)
- ◯ Pregnancy
- ◯ Homelessness
- ◯ Poverty
- ◯ Food/nutrition (including obesity/hunger)
- ◯ Low education
- ◯ Other, please specify:

Please specify other: ______________________________

How important is it for your facility to offer preventive health services, on a scale of 1 to 5 with 1 being least important and 5 most important?

	1- Not at all important	2- Not very important	3- Neutral	4- Somewhat important	5- Very important	Declined to answer
Childhood immunizations	○	○	○	○	○	○
Flu shots	○	○	○	○	○	○
HPV vaccination	○	○	○	○	○	○
STD testing, not including HIV	○	○	○	○	○	○
HIV testing	○	○	○	○	○	○
Pap smears for cervical cancer screening	○	○	○	○	○	○
Birth control for women	○	○	○	○	○	○
Substance abuse treatment	○	○	○	○	○	○
Mental health treatment	○	○	○	○	○	○

Are you able to provide any of the following preventive health information related to sexual health? (Select all that apply)

- ☐ Basic sexual activity information (oral, anal, vaginal, penis)
- ☐ Female reproductive information
- ☐ Birth control information
- ☐ Condom information
- ☐ HPV-cancer information
- ☐ HIV/AIDS information
- ☐ Safer sex education
- ☐ Other, please specify:
- ☐ Declined to answer

Please specify other: ______________________________

Are you able to provide any of the following preventive health services related to sexual health? (Select all that apply)

- ☐ Oral contraception
- ☐ Implants, e.g. Nexplanon
- ☐ Vaginal rings
- ☐ Depo-provera
- ☐ Intrauterine devices/IUDs
- ☐ Contraceptive patches
- ☐ Tubal ligations
- ☐ Vasectomies
- ☐ Condom provision
- ☐ Sexually transmitted infection treatment
- ☐ Other, please specify
- ☐ Declined to answer

Please specify other: ______________________________

Are you able to provide any of the following pregnancy-related services? (Select all that apply)

- ☐ Pregnancy testing
- ☐ Pregnancy options counseling
- ☐ Abortion
- ☐ Prenatal education
- ☐ Prenatal vitamins
- ☐ Comprehensive prenatal care
- ☐ Birth/delivery services
- ☐ Lactation consultant
- ☐ Newborn nursery
- ☐ Other, please specify:
- ☐ Declined to answer

Please specify:

____

What type of substance abuse treatment does your facility provide? (Select all that apply)

- ☐ None
- ☐ NA/AA/12 Step Program
- ☐ Methadone
- ☐ Bupronophrenion
- ☐ Substance abuse counseling
- ☐ Tobacco cessation counseling
- ☐ Other, please specify:
- ☐ Declined to answer

Please specify:

____

What type of mental health services does your facility provide? (Select all that apply)

- ☐ Individual counseling
- ☐ Group counseling
- ☐ Prescription medicines for mental health diagnoses
- ☐ Mental health isolation
- ☐ Other, please specify:
- ☐ Declined to answer

Please specify other:

____

Which, if any, vaccines do you provide to inmates? (Select all that apply)

- ☐ None
- ☐ Flu/influenza
- ☐ DPT/Tap
- ☐ DT
- ☐ Pneumovax
- ☐ HPV
- ☐ Hepatitis B
- ☐ Other, please specify:
- ☐ Declined to answer

Please specify other: __________________________________

If you provide vaccines to inmates, where do vaccination records go? (Select all that apply)

- ☐ Do not provide any vaccines
- ☐ Stay in facility
- ☐ Forwarded to local health department
- ☐ Forwarded to state health registry
- ☐ Other, please specify:
- ☐ Declined to answer

Please specify: __________________________________

Do you have records of past immunizations for inmates? (Select one answer)

- ○ No
- ○ Yes; if yes, please describe the system for accessing these records
- ○ Declined to answer

Please describe:

____

If you were to provide vaccinations to inmates, or you already do, what are some challenges you face or would face? (Select all that apply)

- ☐ Cost
- ☐ Medical staffing available
- ☐ Too many other needs
- ☐ Not a top priority
- ☐ Not our responsibility, as a correctional facility
- ☐ Short length of stays for inmates
- ☐ Other, please specify:
- ☐ Declined to answer

Please specify: ______________________________

If you were to provide HPV VACCINATION to inmates/or you already do, what are some challenges you face or would face? (Select all that apply)

- ☐ Cost
- ☐ Medical staffing available
- ☐ Correctional staffing available
- ☐ Not a top priority
- ☐ Not our responsibility, as a correctional facility
- ☐ Short length of stays for inmates
- ☐ Age of inmates
- ☐ Storage of the vaccine
- ☐ Other, please specify:
- ☐ Declined to answer

Please specify: ______________________________

If you were to offer HPV VACCINATION at your facility, how would you let inmates know about the availability? (Select all that apply)

- ☐ Sign-up sheet in housing units
- ☐ Notice on the medical cart
- ☐ Notice at the medical window
- ☐ At the time of other medical appointment
- ☐ Word of mouth
- ☐ Announcements in housing units by medical staff
- ☐ Sign-up through case managers
- ☐ Other, please specify:
- ☐ Declined to answer

Please specify: ______________________________

If you were to offer HPV VACCINATION at your facility, what would the security concerns be? (Select all that apply)

- ☐ Personal safety of medical staff administering vaccine
- ☐ Safety of inmates
- ☐ Safety of correctional officers
- ☐ Other, please specify:
- ☐ Declined to answer

Please specify: ______________________________

If you were to offer HPV VACCINATION at your facility, in what space would it be possible to physically administer the vaccine? (Select all that apply)

- ☐ In the housing units in a dedicated station
- ☐ On a medical cart brought to the housing unit
- ☐ Through the medical window
- ☐ In the medical clinic
- ☐ Not sure
- ☐ Other, please specify:
- ☐ Declined to answer

Please specify other: ______________________________

If you were to offer HPV VACCINATION at your facility, who would you want to the administer the vaccine? (Select all that apply)

- ☐ Medical staff at the jail
- ☐ Medical staff from the health department
- ☐ Other, please specify:
- ☐ Declined to answer

Please specify: __________________________

If you were to offer HPV VACCINATION at your facility, how would you pay for it? (Select all that apply)

- ☐ Partnership with the local health department
- ☐ Jail health budget
- ☐ Billing inmate's insurance or Medicaid
- ☐ Vaccines for Children program for inmates age 18 and under
- ☐ Do not know
- ☐ Declined to answer

Is your facility a Vaccines for Children ("VFC") Provider? (Select one answer)

- ◯ Yes
- ◯ No
- ◯ Do not know
- ◯ Declined to answer

How possible would it be for your facility to run an HPV VACCINATION program? (Select one answer)

- ◯ Not at all possible at this time
- ◯ Somewhat possible at this time
- ◯ Quite possible at this time
- ◯ Very possible at this time
- ◯ We have already implemented an HPV vaccination program
- ◯ Declined to answer

How possible would it be for a local health department to come in and run an HPV VACCINATION program at your facility? (Select one answer)

- ◯ Not at all possible at this time
- ◯ Somewhat possible at this time
- ◯ Quite possible at this time
- ◯ Very possible at this time
- ◯ We have already implemented an HPV vaccination program
- ◯ Declined to answer

How ready is your facility for implementing an HPV VACCINATION program with the help of a local health department? (Select one answer)

- ◯ No interest or intention to implement
- ◯ Interested in finding out more about implementation
- ◯ Interested and has some groundwork laid to implement
- ◯ We have already implemented an HPV vaccination program
- ◯ Declined to answer

If your local health department were willing to initiate an HPV VACCINATION program, how soon could you implement the program? (Select one answer)

- ◯ In the next month
- ◯ In the next six months
- ◯ In the next year
- ◯ Could not happen in foreseeable future
- ◯ We have already implemented an HPV vaccination program
- ◯ Declined to answer

What would work in your facility for providing inmates education about HPV VACCINATION? (Select all that apply)

- ☐ Pamphlet with HPV information
- ☐ Posters with HPV information
- ☐ DVD programming with HPV information
- ☐ Internet-based information for an iPad or some other electronic device
- ☐ Education for medical staff
- ☐ Education for correctional staff
- ☐ Health education from local health department staff
- ☐ Other, please specify:
- ☐ Declined to answer

Please specify: __________________________________

If local health department staff were to administer an HPV VACCINATION program, what would be jail staffing needs? (Select all that apply)

- ☐ Correctional officers available to escort/guard health department staff
- ☐ Medical staff supervision/coordination
- ☐ Other, please specify:
- ☐ We have already implemented an HPV vaccination program
- ☐ Declined to answer

Please specify: __________________________________

If your facility were to work with your local health department to offer an HPV VACCINATION clinic, how often would the clinic be available? (Select one answer)

- ◯ 5 days per week
- ◯ One per week
- ◯ Once per month
- ◯ 2-4 times per year
- ◯ No time to do this
- ◯ Other, please specify:
- ◯ We have already implemented an HPV vaccination program
- ◯ Declined to answer

Please specify: __________________________________

If we wanted to help you facilitate an HPV VACCINATION clinic, who would be the best person with whom to work? (Select one answer)

- ◯ Warden or lead administrator
- ◯ Head of health services
- ◯ Nurse in health services
- ◯ No one
- ◯ Other, please specify:
- ◯ Declined to answer

Please specify: __________________________________

If your facility serves juveniles, how is consent obtained for the health care of juveniles in your facility?

- ◯ From their parents or guardians
- ◯ Jail health staff act as guardians for decision-making
- ◯ Other, please explain:
- ◯ Our facility does not serve juveniles
- ◯ Declined to answer

Please explain: __________________________________
