# Supplementary material for: Collaborating to offer HPV vaccinations in jails: results from a pre-implementation study in four states
Source: BMC Health Serv Res. 2021 Apr 7;21:309. doi: 10.1186/s12913-021-06315-5 (PMC8028758; doi:10.1186/s12913-021-06315-5)
Supplement: Supplementary file 2 — Additional file 2. Health Department Survey about Collaborating with Local Jails for HPV Vaccination. [file 12913_2021_6315_MOESM2_ESM.pdf]

Health Department Survey about Collaborating with Local Jails for HPV Vaccination

Thank you so much for participating in this survey about concerns and ideas you may have about partnering with local jails to help incarcerated young adults (aged 18-26) and youth (aged 9-17) access the human papillomavirus (HPV) vaccination series.

As a reminder, the HPV vaccine prevents several kinds of cancer, and is recommended to be offered with other routine vaccines. We are trying to assess if partnerships between county jails and health departments might serve the HPV vaccine needs of inmates.

This survey should take about 10 minutes. It is anonymous. You can stop at any time or decline to answer any questions.

Thank you!

In which state is your health department located? (Select one answer)

- ◯ Kansas
- ◯ Missouri
- ◯ Iowa
- ◯ Nebraska
- ◯ Declined to answer

What county(ies) does your health department serve? (Please list all)

____

How many overall employees does your health department have?

____

How many patients do you serve per year (all)?

____

What is the approximate percent of patients who are under 18?

____

What is the approximate percent of patients who are racial or ethnic minorities?

____

What is the approximate percent of patients who are uninsured?

____

How many days per month (0-30) is routine medical care available with these providers at your health department? (If your health department does not have a particular provider, please specify with a "0")

General physicians: ______________________ (DAYS PER MONTH)

OBGYNs: ______________________ (DAYS PER MONTH)

Mental health providers: ______________________ (DAYS PER MONTH)

Other specialists (please specify titles below): ______________________ (DAYS PER MONTH)

Please specify other specialists: ______________________

Nurse practitioners/ Physician's assistants ______________________ (DAYS PER MONTH)

Registered nurses: ______________________ (DAYS PER MONTH)

LPNs (licensed practical nurses) or equivalent: ______________________ (DAYS PER MONTH)

Medical assistants (non-licensed): ______________________ (DAYS PER MONTH)

Who is in charge of Immunization Services at your health department? (Select all that apply)

- ☐ Physician
- ☐ Nurse practitioner
- ☐ Physician's assistant
- ☐ Registered nurse
- ☐ LPN
- ☐ Other, please specify:
- ☐ Declined to answer

Please specify other: ______________________

Does your health department bill third-party payers for health services? (Select one answer)

- ◯ Yes
- ◯ No
- ◯ Do not know
- ◯ Declined to answer

Are you able to provide any of the following preventive health information related to sexual health? (Select all that apply)

- ☐ Basic sexual activity information (oral, anal, vaginal, penis)
- ☐ Female reproductive information
- ☐ Birth control information
- ☐ Condom information
- ☐ HPV-cancer information
- ☐ HIV/AIDS information
- ☐ Safer sex education
- ☐ Other, please specify:
- ☐ Declined to answer

Please specify other: ______________________

Are you able to provide any of the following preventive health services related to sexual health? (Select all that apply)

- ☐ Oral contraception
- ☐ Implants, e.g. Nexplanon
- ☐ Vaginal rings
- ☐ Depo-provera
- ☐ Intrauterine devices/IUDs
- ☐ Contraceptive patches
- ☐ Tubal ligations
- ☐ Vasectomies
- ☐ Condom provision
- ☐ Sexually transmitted infection treatment
- ☐ Other, please specify
- ☐ Declined to answer

Please specify other: ______________________________

Are you able to provide any of the following pregnancy-related services? (Select all that apply)

- ☐ Pregnancy testing
- ☐ Pregnancy options counseling
- ☐ Abortion
- ☐ Prenatal education
- ☐ Prenatal vitamins
- ☐ Comprehensive prenatal care
- ☐ Birth/delivery services
- ☐ Lactation consultant
- ☐ Newborn nursery
- ☐ Other, please specify
- ☐ Declined to answer

Please specify other: ______________________________

Does your health department offer any of the following substance abuse treatments? (Select all that apply)

- ☐ None
- ☐ NA/AA/12 Step Program
- ☐ Methadone
- ☐ Buprenorphine
- ☐ Substance abuse behavioral health
- ☐ Tobacco cessation counseling
- ☐ Other, please specify:
- ☐ Declined to answer

Please specify other: ______________________________

Does your health department offer any of the following mental health treatments? (Select all that apply)

- ☐ Individual counseling/therapy
- ☐ Group counseling/therapy
- ☐ Family counseling/therapy
- ☐ Prescription medicines for mental health diagnoses
- ☐ Other, please specify:
- ☐ Declined to answer

Please specify other: ______________________________

Which vaccines does your health department provide? (Select all that apply)

- ☐ None
- ☐ Flu/Influenza
- ☐ DPT/Tap
- ☐ DT
- ☐ Pneumovax
- ☐ HPV
- ☐ Hepatitis B
- ☐ Other, please specify:
- ☐ Declined to answer

Please specify other: ______________________________

How is consent obtained for the health care of youth under age 18? (Check one answer)

- ◯ From their parents or guardians
- ◯ Youth under age 18 are able to consent to sexual health services
- ◯ Youth under age 18 are able to consent for HPV vaccination
- ◯ Other, please explain:
- ◯ Declined to answer

Please explain other: ____

How important do you think it is for your COMMUNITY'S JAIL to offer preventive health services, on a scale of 1 to 5, with 1 being least important and 5 most important?

	1- Not at all important	2- Not very important	3- Neutral	4- Somewhat important	5- Very important	Declined to answer
Childhood immunizations	◯	◯	◯	◯	◯	◯
Flu shots	◯	◯	◯	◯	◯	◯
HPV vaccination	◯	◯	◯	◯	◯	◯
STD testing, not including HIV	◯	◯	◯	◯	◯	◯
HIV testing	◯	◯	◯	◯	◯	◯
Pap smears for cervical cancer screening	◯	◯	◯	◯	◯	◯
Birth control for women	◯	◯	◯	◯	◯	◯
Substance abuse treatment	◯	◯	◯	◯	◯	◯
Mental health treatment	◯	◯	◯	◯	◯	◯

Think about the people INCARCERATED IN YOUR COMMUNITY'S LOCAL JAIL. What do you think are the top three most important health issues of inmates in your community's jail?

Most important:

◯ Lack of health insurance in community
◯ Lack of regular health care
◯ Lack of primary care provider
◯ Lack of vaccinations
◯ Lack of health knowledge
◯ Substance use
◯ Mental health issues
◯ Chromic disease (example: asthma, diabetes, or heart disease)
◯ Infectious disease (example: HIV, sexually transmitted infections, or Hepatitis C)
◯ Pregnancy
◯ Homelessness
◯ Poverty
◯ Food/nutrition (including obesity/hunger)
◯ Low education
◯ Other, please specify:

Please specify other: ______________________________

Second most important:

- ◯ Lack of health insurance in community
- ◯ Lack of regular health care
- ◯ Lack of primary care provider
- ◯ Lack of vaccinations
- ◯ Lack of health knowledge
- ◯ Substance use
- ◯ Mental health issues
- ◯ Chromic disease (example: asthma, diabetes, or heart disease)
- ◯ Infectious disease (example: HIV, sexually transmitted infections, or Hepatitis C)
- ◯ Pregnancy
- ◯ Homelessness
- ◯ Poverty
- ◯ Food/nutrition (including obesity/hunger)
- ◯ Low education
- ◯ Other, please specify:

Please specify other: __________________________________

Third most important:

- ◯ Lack of health insurance in community
- ◯ Lack of regular health care
- ◯ Lack of primary care provider
- ◯ Lack of vaccinations
- ◯ Lack of health knowledge
- ◯ Substance use
- ◯ Mental health issues
- ◯ Chromic disease (example: asthma, diabetes, or heart disease)
- ◯ Infectious disease (example: HIV, sexually transmitted infections, or Hepatitis C)
- ◯ Pregnancy
- ◯ Homelessness
- ◯ Poverty
- ◯ Food/nutrition (including obesity/hunger)
- ◯ Low education
- ◯ Other, please specify:

Please specify other: __________________________________

Does your health department provide any vaccines to INMATES IN YOUR LOCAL CORRECTIONAL FACILITIES? (Select all that apply)

- ☐ No
- ☐ Yes, to patients under 18
- ☐ Yes, to patients 18 and over
- ☐ Declined to answer

If you provide vaccines to INMATES, where do vaccination records go? (Select all that apply)

- ☐ Do not provide any vaccines
- ☐ Stay in health department
- ☐ Forwarded to local jail
- ☐ Forwarded to state health registry
- ☐ Other, please specify:
- ☐ Declined to answer

Please specify other: __________________________________

If you were to provide vaccinations to INMATES IN YOUR LOCAL CORRECTIONAL FACILITY, or you already do, what are some challenges you face or would face? (Select all that apply)

- ☐ Cost
- ☐ Medical staffing available
- ☐ Correctional staffing available
- ☐ Too many other needs
- ☐ Not a top priority
- ☐ Not our responsibility as a health department
- ☐ Short length of stays for inmates
- ☐ Storage of vaccine
- ☐ Other, please specify:
- ☐ Declined to answer

Please specify other: __________________________________

If you were to offer HPV VACCINATION AT YOUR LOCAL CORRECTIONAL FACILITY, who would you want to administer the vaccine? (Select one answer)

- ○ Medical staff at the jail
- ○ Medical staff from the health department
- ○ Other, please specify:
- ○ Declined to answer

Please specify other: __________________________________

If you were to offer HPV VACCINATION AT YOUR LOCAL CORRECTIONAL FACILITY, how would you pay for it? (Select all that apply)

- ☐ Health department budget
- ☐ Jail health budget
- ☐ Billing inmate's insurance of Medicaid
- ☐ Vaccines for Children program
- ☐ Pharmaceutical patient assistance program
- ☐ Do not know
- ☐ Declined to answer

How possible would it be for your health department to run an HPV VACCINATION PROGRAM IN YOUR LOCAL CORRECTIONAL FACILITY? (Select one answer)

- ◯ Not at all possible at this time
- ◯ Somewhat possible at this time
- ◯ Quite possible at this time
- ◯ Very possible at this time
- ◯ Declined to answer

How ready is your health department to implement an HPV vaccination program AT YOUR LOCAL CORRECTIONAL FACILITY? (Select one answer)

- ◯ No interest or intention to implement
- ◯ Interested in finding out more about implementation
- ◯ Interested and has some groundwork laid to implement
- ◯ Already have HPV vaccine
- ◯ Declined to answer

If your local correctional facility were willing to initiate an HPV VACCINATION PROGRAM with your health department, how soon could you implement the program? (Select one answer)

- ◯ In the next month
- ◯ In the next six months
- ◯ In the next year
- ◯ Could not happen in the foreseeable future
- ◯ Already implemented
- ◯ Declined to answer

What might work for you to provide INMATES with education about HPV vaccination? (Check all that apply)

- ☐ Pamphlet with HPV information
- ☐ Posters with HPV information
- ☐ DVD programming with HPV information
- ☐ Internet-based information for an iPad or some other electronic device
- ☐ Training for medical staff
- ☐ Training session for correctional staff
- ☐ Health education from our health department staff
- ☐ Other, please specify:
- ☐ Declined to answer

Please specify other:

____

What security concerns do you have about your health department running a vaccine program AT YOUR LOCAL CORRECTIONAL FACILITY? (Check all that apply)

- ☐ Ensuring health department staff appreciate security requirements at the jail
- ☐ Approving health department staff for jail access
- ☐ Inmate issues
- ☐ Other, please specify:
- ☐ Declined to answer

Please specify other:

____

If your health department were to work with your LOCAL CORRECTIONAL FACILITY to offer an HPV vaccination clinic, how often would the clinic be available? (Check one answer)

- ◯ 2-4 times per year
- ◯ Once per month
- ◯ Once per week
- ◯ 5 days per week
- ◯ No time to do this
- ◯ Other, please specify:
- ◯ Declined to answer

Please specify other: __________________________________

If we wanted to help you facilitate an HPV vaccination clinic at your LOCAL CORRECTIONAL FACILITY, who would be the best person with whom to work? (Check one answer)

- ◯ Medical staff at the jail
- ◯ Medical staff from the health department
- ◯ Other, please specify:
- ◯ Declined to answer

Please specify other: ______________________________
