# Supplementary material for: Collaborating to offer HPV vaccinations in jails: results from a pre-implementation study in four states
Source: BMC Health Serv Res. 2021 Apr 7;21:309. doi: 10.1186/s12913-021-06315-5 (PMC8028758; doi:10.1186/s12913-021-06315-5)
Supplement: Supplementary file 3 — Additional file 3. Open-ended Questions. [file 12913_2021_6315_MOESM3_ESM.docx]

**Additional File 3**

*Open-ended Questions for Jail and LHD Respondents Answering “Already Have” or “Have Plans for” a Vaccination Program” in the Jail*

1. Can you walk me through how offering the HPV vaccine to inmates at the jail came about?
2. Who was the person/people (or job titles) that initiated initiate the relationship between your health department and local jail?
3. What made it possible to start providing HPV vaccinations to inmates?
4. (Depending on who initiated initiated the relationship) What was the initial response from your LHD/local jail in being asked if HPV vaccinations could be offered to inmates?
5. Who at your jail/health department was responsible for communicating with your local jail/LHD to make sure HPV vaccinations could be offered/given to inmates? How regularly did/do they communicate? Who at the jail/LHD do they speak with?
6. Who at the jail/health department is responsible for giving HPV vaccinations to inmates?
7. Where are vaccinations given?
8. How often are HPV vaccinations offered at the jail (or at the HD if inmates are brought over)?
9. How do inmates pay for the vaccination?
10. What is the consent process for inmates, particularly if patients are under 18?
11. How does your health department follow up with vaccinated patients from the jail for more doses if needed?
12. How is information about the vaccine provided provided to inmates?
